# Supplementary material for: Targeted systematic evolution of an RNA platform neutralizing DNMT1 function and controlling DNA methylation
Source: Nat Commun. 2023 Jan 6;14:99. doi: 10.1038/s41467-022-35222-4 (PMC9823104; doi:10.1038/s41467-022-35222-4)
Supplement: Supplementary file 1 — Supplementary Information [file 41467_2022_35222_MOESM1_ESM.pdf]

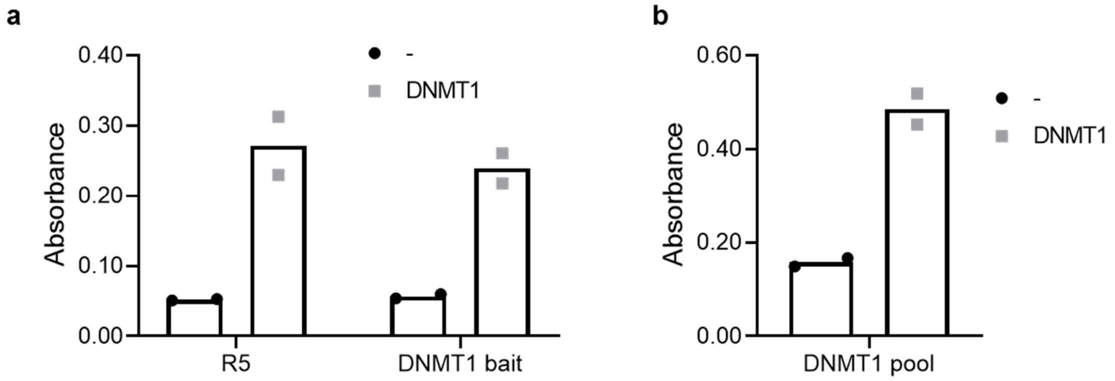

Supplementary Figure 1. Binding of the DNMT1 bait and the enriched library on DNMT1 purified protein. (**a**) Binding ability of umodified R5 sequence (R5) or R5 modified with 2'-FPy (DNMT1 bait) on DNMT1 purified protein was detected by ELONA. (**b**) Binding ability of the enriched library after 3 rounds of selection (DNMT1 pool) on DNMT1 purified protein detected by ELONA. In (**a, b**) Experiments are representative of two independent biological replicates with similar results (n=2). Mean and the corresponding data points are reported.

```
                              10        20
                     ....|....|....|....|..
Ce-37                CUGAGGCCUUGUCUAGGCUUCU
Ce-41                CUGAGGUCUUGUCGAGGCAUCC
Ce-44                CUGAGGCCUUGGCGAGGCAUCC
Ce-11                CUGAGGCCUUGGCGAGGCGACC
Ce-39                CUGAGGCCUUGGCGAGGCUCCC
Ce-54                CUGAGGCCUUGGCGAGGCGCCC
Ce-32                CUGAGGCCUUGGCGAGGCACCC
Ce-36                CUGAGUGCUUGGCGAGACUUCU
Ce-1                 CUGAGCGACUGGCGAGGCUUCU
Ce-8                 CUGAGUAACUGGCGAGGCUUCU
Ce-27                CUGAGCAACUGGCGAGGCUUCU
Ce-28                CUGAGUGCCUGGCGAGGCUUCU
Ce-65                CUGAGUACCUGGCGAGGCUUCU
Ce-68                CUGAGGCCUUGGCGAGGCAGUU
Ce-14                CUGAGGCCUUGGCGAGGCUGAC
Ce-13                CUGAGGCCUUGGCGAGGCGGAC
Ce-16                CUGAGGCCUUGGCGAGGCCGUC
Ce-63                CUGAGGCCUUGGCGAGGCCCGC
Ce-57                CUGAGGCCUUGGCGAGGCACGU
Ce-64                CUGAGGCCUUGGCGAGGCUUGU
Ce-17                CUGAGGCCUUGGCGAGGCUCGU
Ce-42                CUGAGACCGUGGCGAGGCUUCU
Ce-59                CUGAGCCCGUGGCGAGGCUUCU
Ce-60                CUGAGGCCUUGGCGAGGCUCUG
Ce-61                CUGAGGCCUUGGCGAGGCGCUG
Ce-2                 CUGAGUUGCUGGCGAGGCUUCU
Ce-3                 CUGAGUAUAUGGCGAGGCUUCU
Ce-20                CUGAGUUAUUGGCGAGGCUUCU
Ce-52                CUGAGCAGGUGGCGAGGCUUCU
Ce-33                CUGAGAACCUGGCGAGGCUUCC
Ce-15                CUGAGAACUUGGCGAGGCUUCU
Ce-67                CUGAGAAGAUGGCGAGGCUUCU
Ce-6                 CUGAGCCCUUGGCGAGGCUUCC
Ce-50                CUGAGACCUUGGCGAGGCUUCC
Ce-62                CUGAGGCCUUGGCGAGGCCUGG
Ce-66                CUGAGGCCUUGGCGAGGCCAAA
Ce-21                CUGAGGCCUUGGCGAGGCCACG
Ce-43                CUGAGGCCUUGGCGAGGCAACG
Ce-5                 CUGAGGCCUUGGCGAGGCUUCG
Ce-29                CUGAGGCAUUGGCGAGGCUUCU
Ce-10                CUGAGGUAAUGGCGAGGCUUCU
Ce-51                CUGAGGUAAUGGCGAGGCUUCU
Ce-9                 CUGAGCUCAUGGCGAGGCUUCU
Ce-22                CUGAGCUCAUGGCGAGGCUUCU
Ce-56                CUGAGGACAUGGCGAGGCUUCU
Ce-49                CUGAGGCCUAACGAAGGCUUCU
Ce-23                CUGAGGCCUACCCAAGGCUUCU
Ce-38                CUGAGGCCUAGCCAAGGCUUCU
Ce-46                CUGAGGCCUAGCACAGGCUUCU
Ce-70                CUGAGGCCUAGCAAAGGCUUCU
Ce-58                CUGAGGCCUGAUGUAGGCUUCU
Ce-18                CUGAGGCCUGGUGGAGGCUUCU
Ce-69                CUGAGGCCUACUGGAGGCUUCU
Ce-4                 CUGAGGCCUACACUAGGCUUCU
Ce-7                 CUGAGGCCUGCAGUAGGCUUCU
Ce-30                CUGAGGCCUGCCCUAGGCUUCU
Ce-47                CUGAGGCCUGCCGUAGGCUUCU
Ce-26                CUGAGGCCUCAGCUAGGCUUCU
Ce-40                CUGAGGCCUGAACAAGGCUUCU
Ce-45                CUGAGGCCUCCAUAAGGCUUCU
Ce-48                CUGAGGCCUGGGUCAGGCUUCU
Ce-53                CUGAGGCCUGAGUCAGGCUUCU
Ce-24                CUGAGGCCUAUGUAAGGCUUCU
Ce-25                CUGAGGCCUAUGUGAGGCUUCU
Ce-34                CUGAGGCCUAUGGUAGGCUUCU
Clustal Consensus    *****         ** *
```

Supplementary Figure 2. Allignment of individual aptamers from SELEX. Alligment by *Muscle* Algorithm of individual clones from SELEX.

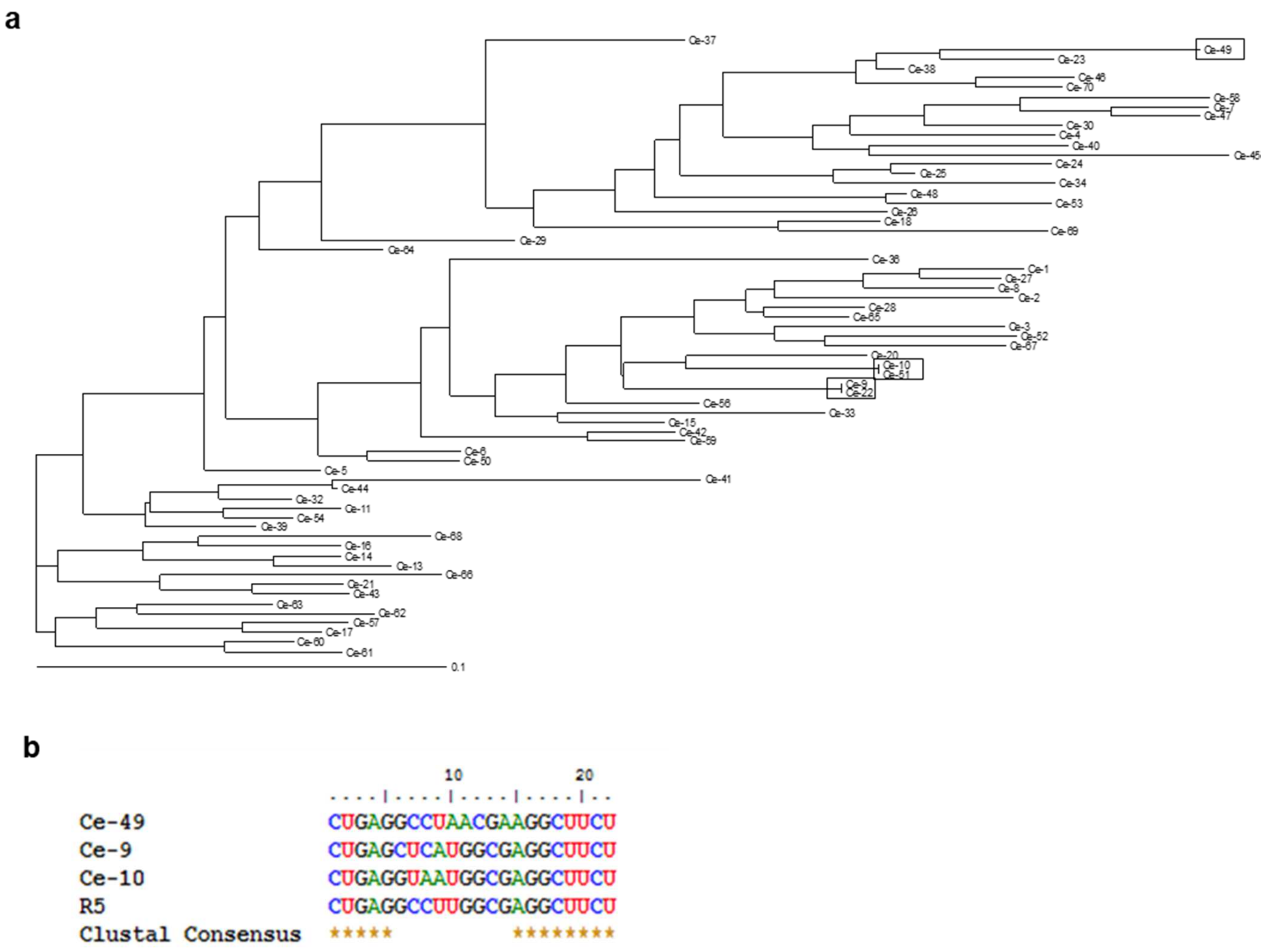

Supplementary Figure 3. Families of individual aptamers from SELEX. (**a**) Dendrogram of the individual sequences cloned after the SELEX rounds. The three sequences chosen for further analyses are boxed. (**b**) Alignment of the central sequences of the three selected aptamers from SELEX (Ce-49; Ce-9 and Ce-10) and R5.

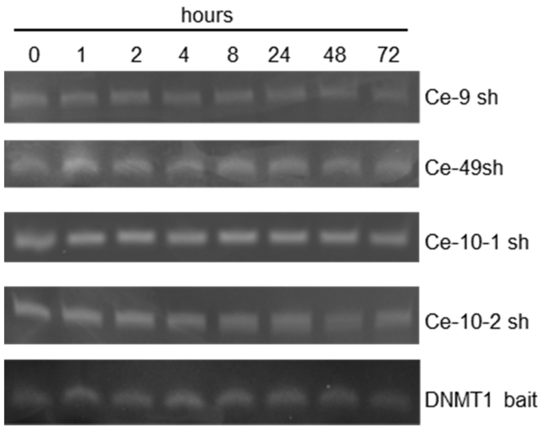

Supplementary Figure 4. Serum stability of short individual aptamers. Short aptamers and DNMT1 bait serum stability were measured in 85% human serum for indicated times. At each time point, RNA-serum samples were collected and evaluated by electrophoresis with 15% denaturing polyacrylamide gel. Gels were stained with ethidium bromide. Experiment was repeated independently three times with similar results.

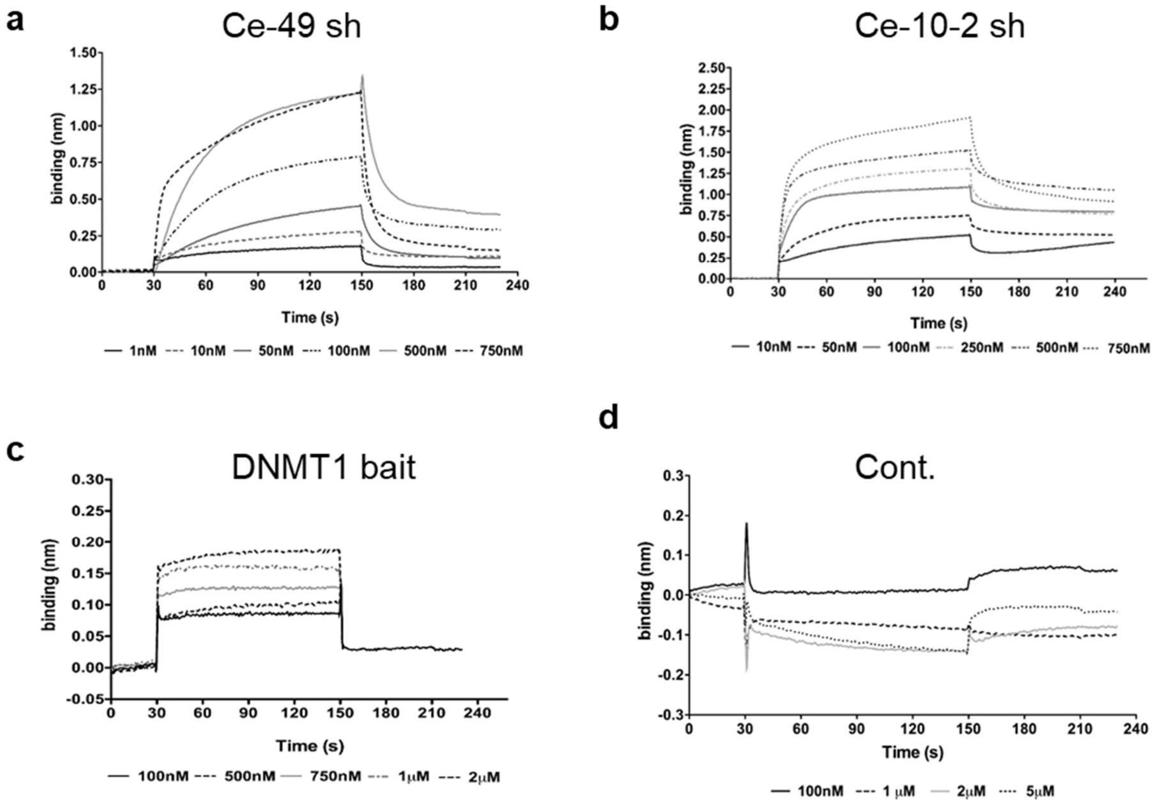

Supplementary Figure 5. BLI affinity analyses. (**a-d**) Bio-Layer Interferometry dose-response measurements of Ce-49 sh (**a**), Ce-10-2 sh (**b**), DNMT1 bait (**c**) or mut-R5 (Cont., used as a negative control) binding to DNMT1 functionalized-biosensors (**d**).

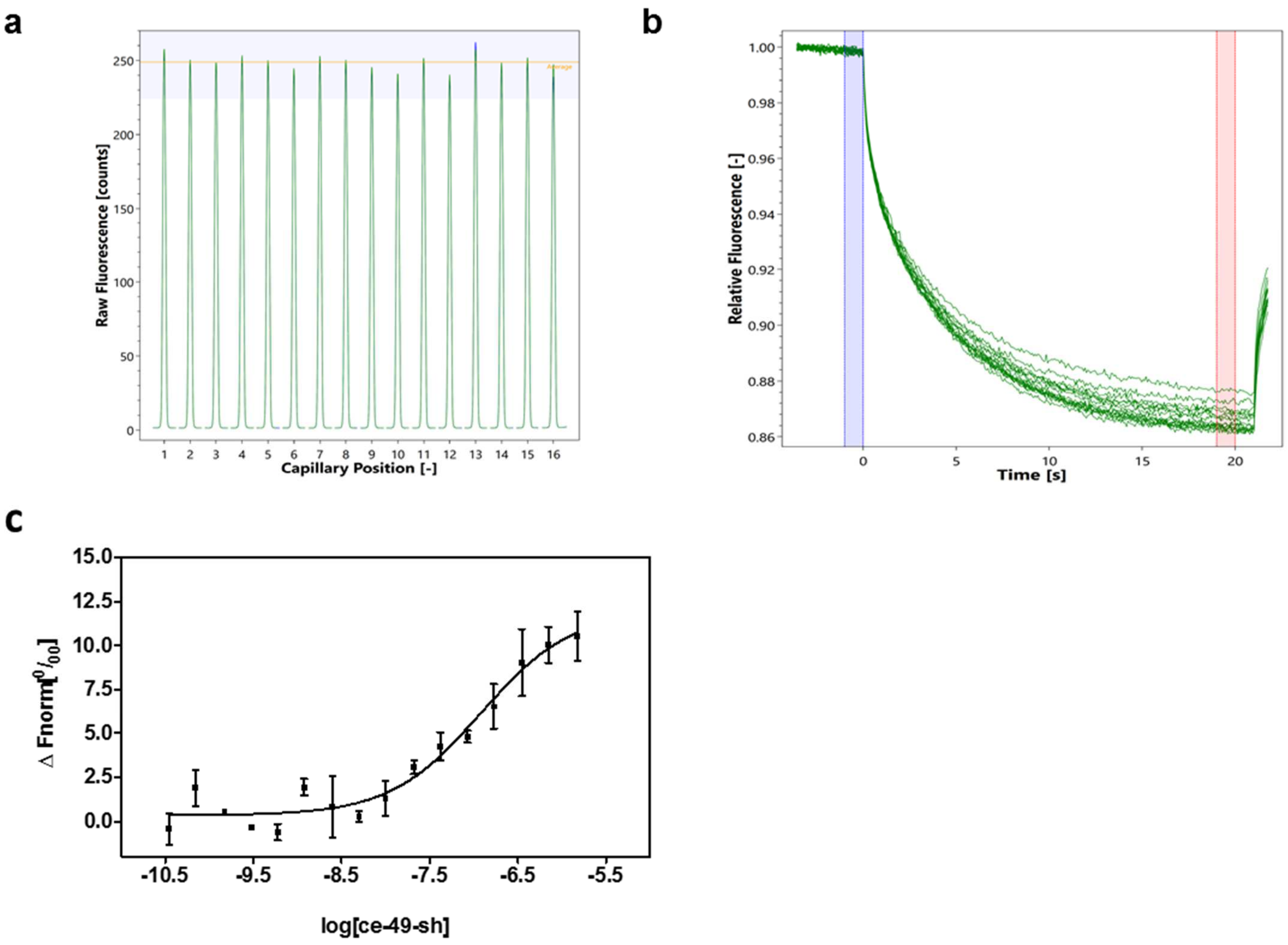

Supplementary Figure 6. MST interaction analysis of Ce-49-sh aptamer against DNMT1. (**a**) Capillary scan, (**b**) MST traces, (**c**) MST dose–response curves reported as ΔFnorm = normalized fluorescence. Means ± SD of two independent replicate (n=3) is reported. A dissociation constants (K_D ± SD) of 119 ± 34 * 10^{-9} M and signal-to-noise ratios (S/N) of 8.719 were calculated using the software MO Affinity Analysis, v 2.27.

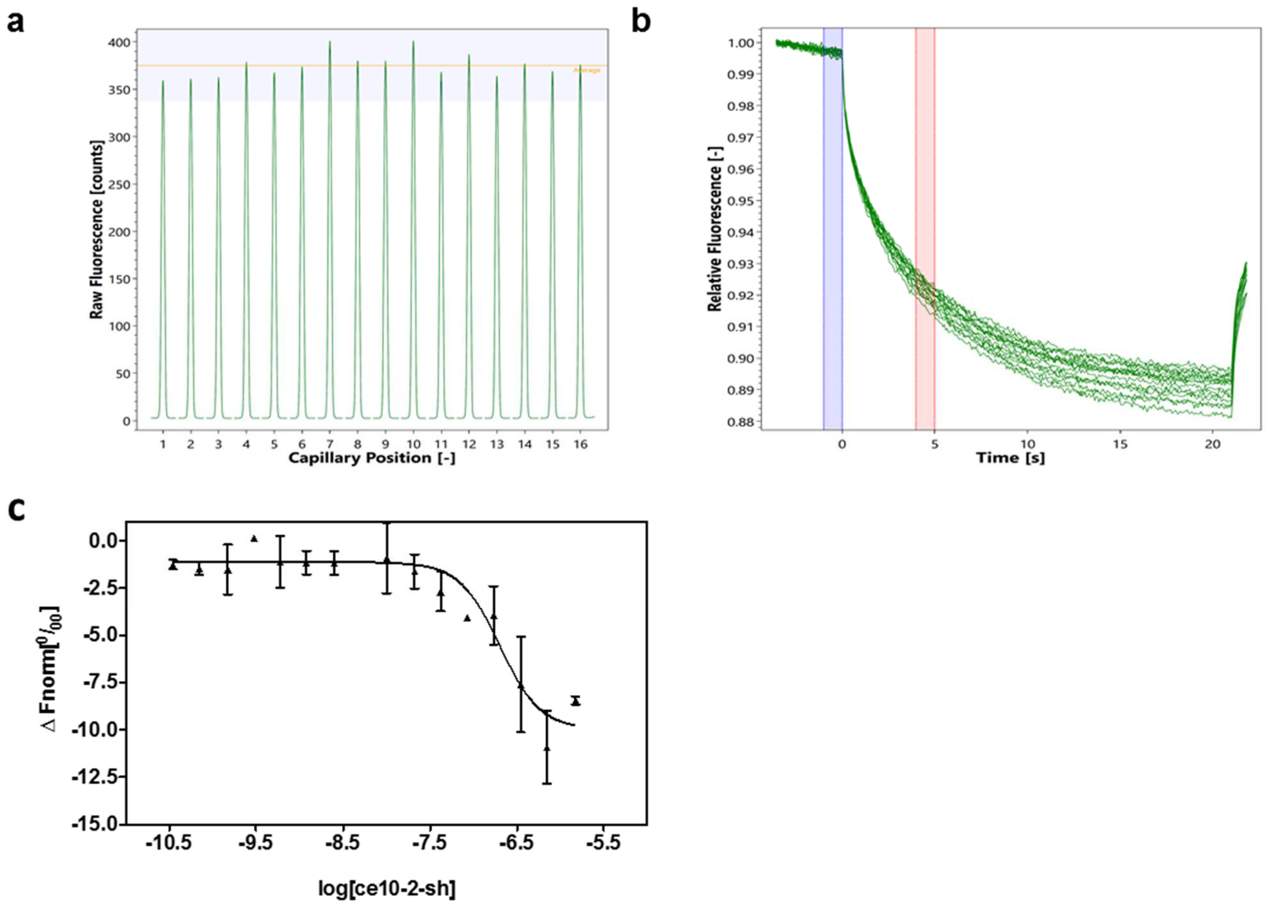

Supplementary Figure 7. Panel of MST interaction analysis of Ce10-2-sh aptamer against DNMT1. (**a**) Capillary scan, (**b**) MST traces, (**c**) MST dose–response curves reported as ΔFnorm = normalized fluorescence. ΔFnorm = normalized fluorescence. Means ± SD of two independent replicate (n=3) is reported. A dissociation constants (K_D ± SD) of 106 ± 42* 10^{-9} M and signal-to-noise ratios (S/N) of 7.685 were calculated by using the MO software Affinity Analysis, v 2.27.

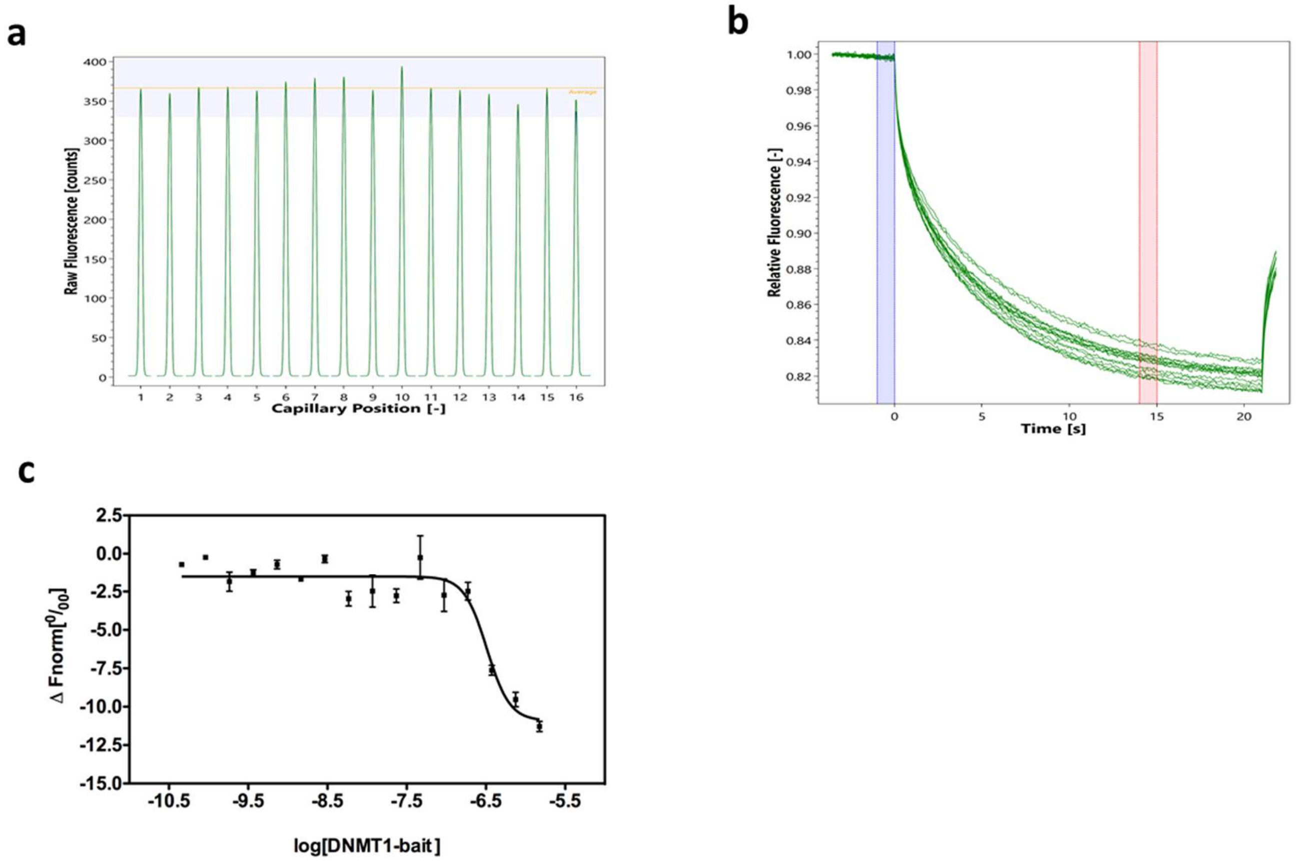

Supplementary Figure 8. Panel of MST interaction analysis of DNMT1 bait against DNMT1. (**a**) Capillary scan, (**b**) MST traces, (**c**) MST dose–response curves reported as ΔFnorm = normalized fluorescence. ΔFnorm = normalized fluorescence. Means ± SD of two independent replicate (n=2) is reported. A dissociation constants (K_D ± SD) of 0.44 ± 0.14 x 10^{-6} M and signal-to-noise ratios (S/N) of 6.508 were calculated by using the MO software Affinity Analysis, v 2.27.

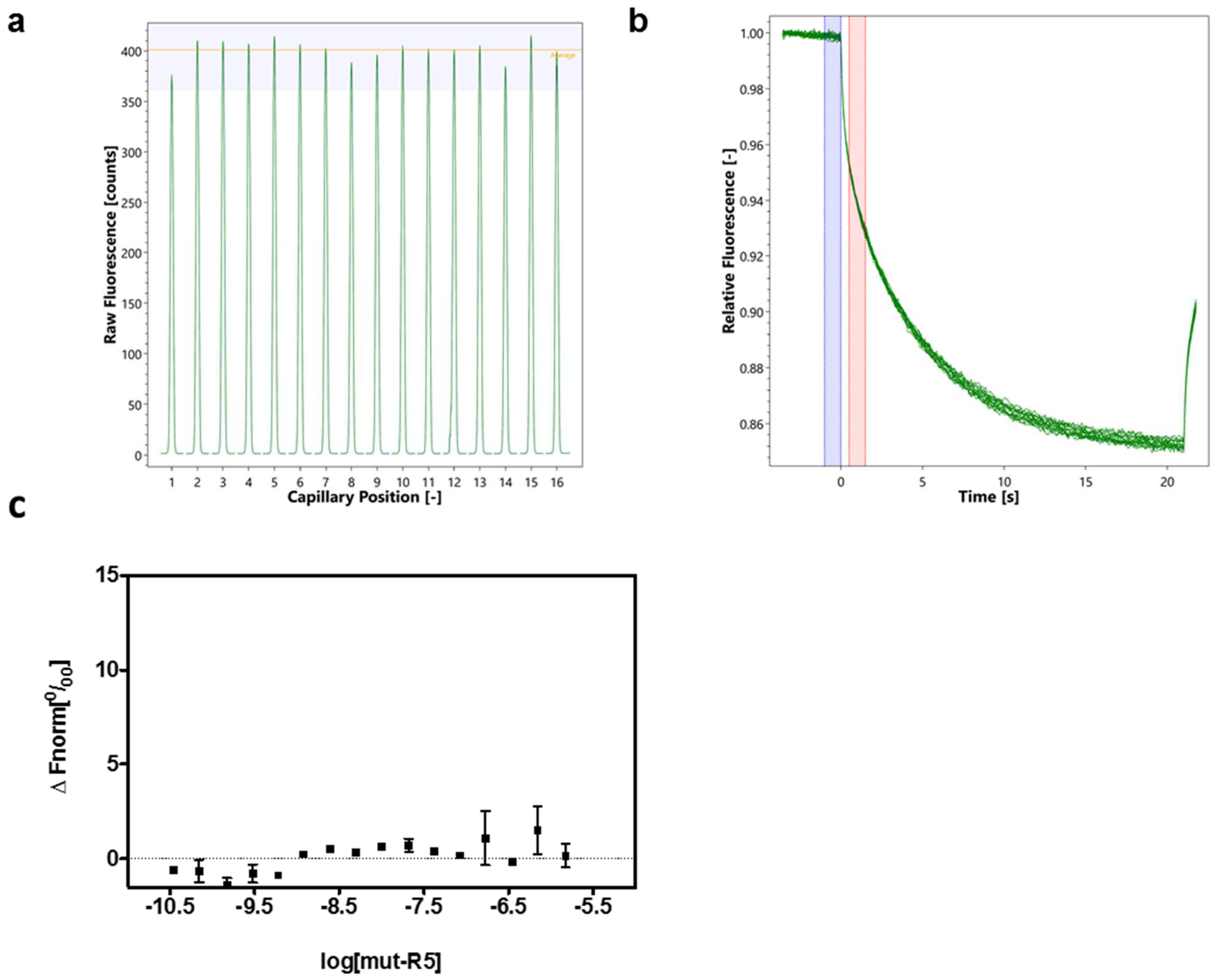

Supplementary Figure 9. Panel of MST interaction analysis of the mut-R5 negative control aptamer against DNMT1. (**a**) Capillary scan, (**b**) MST traces, (**c**) MST dose–response curves reported as ΔFnorm = normalized fluorescence. ΔFnorm = normalized fluorescence. No dose-dependent correlation was detected using the MO software Affinity Analysis, v 2.27. Means ± SD of two independent replicate (n=2) is reported.

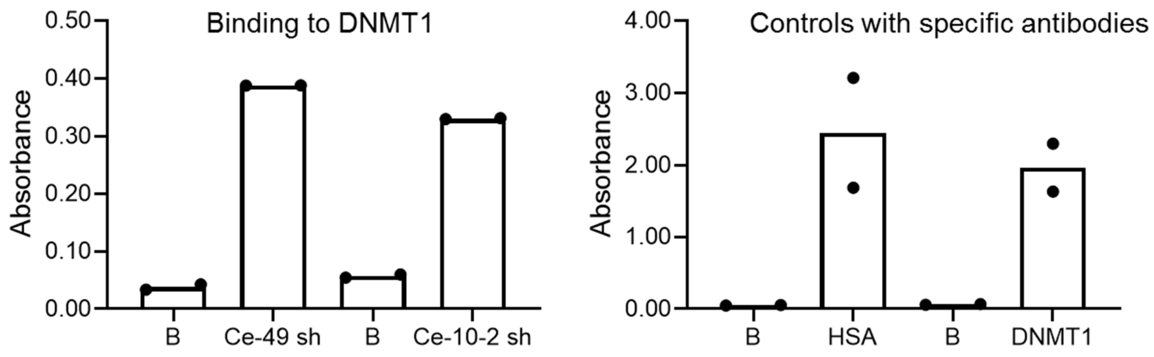

Supplementary Figure 10. Aptamer specificity. For HSA assay, controls were performed by incubating aptamers at 200 nM with DNMT1 protein (*left*) or using specific antibodies to check the effective coating of the plates (*right*). Graphs are representative of two independent biological replicates with similar results (n=2). Mean and the corresponding data points are reported.

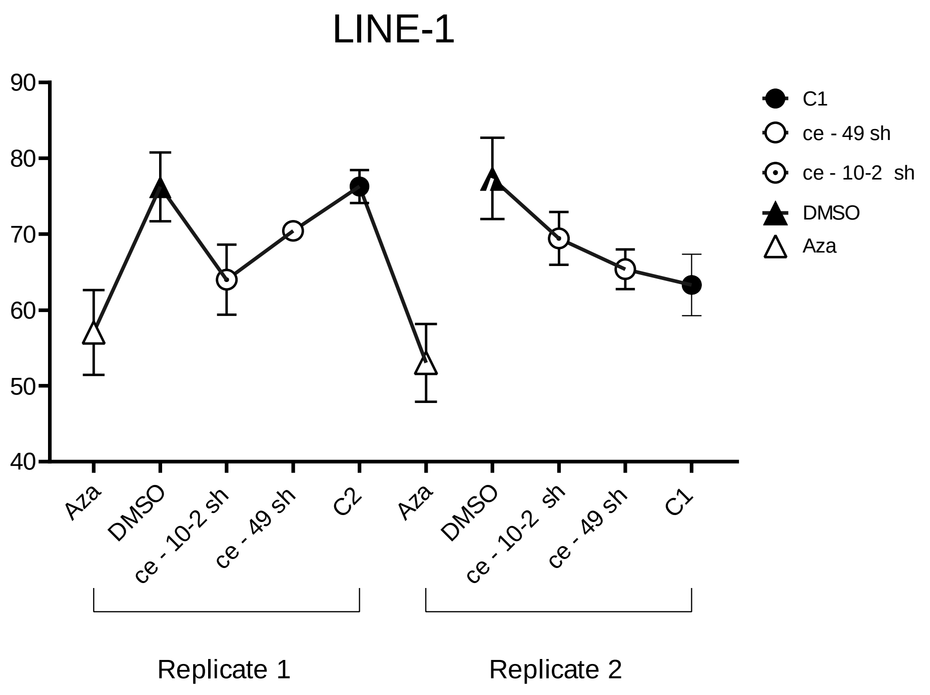

Supplementary Figure 11. LINE 1 analyses. DNA methylation profile of LINE-1 assayed by pyrosequencing in in K562 transfected with Ce-49 sh, or Cont. or treated with DMSO or 5-aza. Mean ± SD is reported.

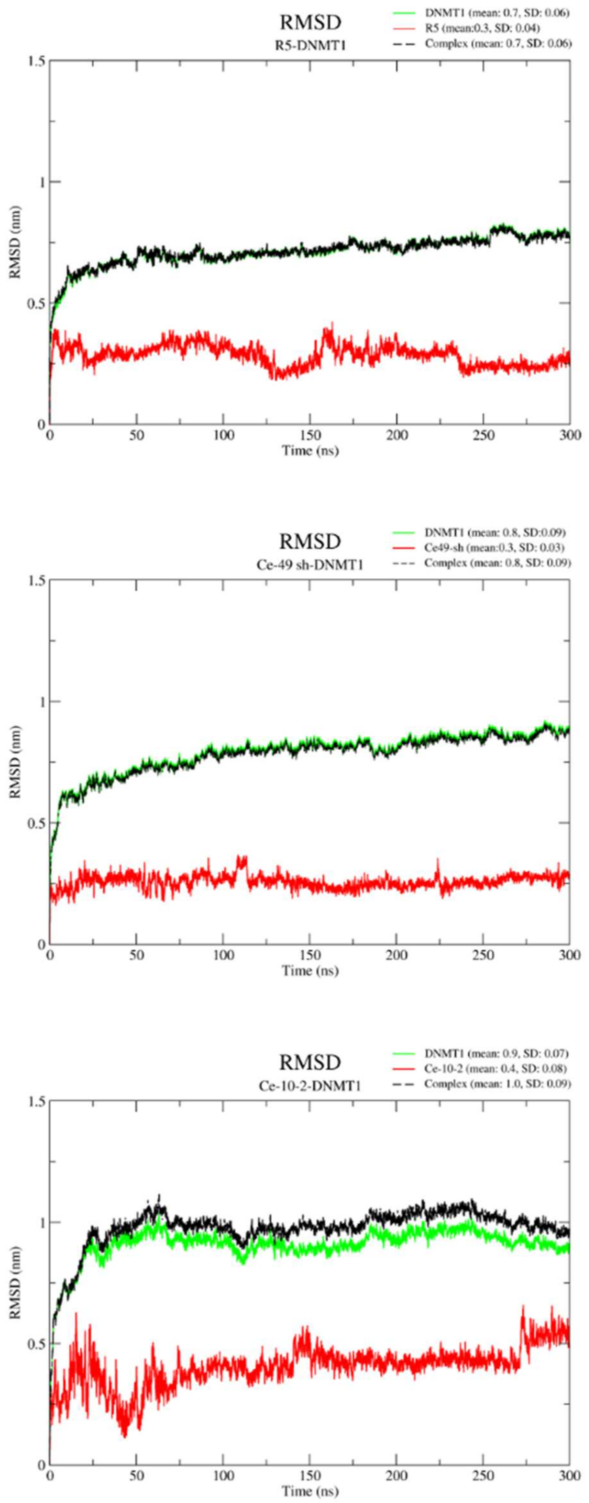

Supplementary Figure 12. Time evolution of the RMSD values respect to the starting models. The RMSD have been computed considering the C alpha and C5’ atoms of the protein and RNA, respectively during the simulations with the amber Parmfsbc1 force field. The following color code was used: overall complexes, black; green, protein residues; red, RNA residues.

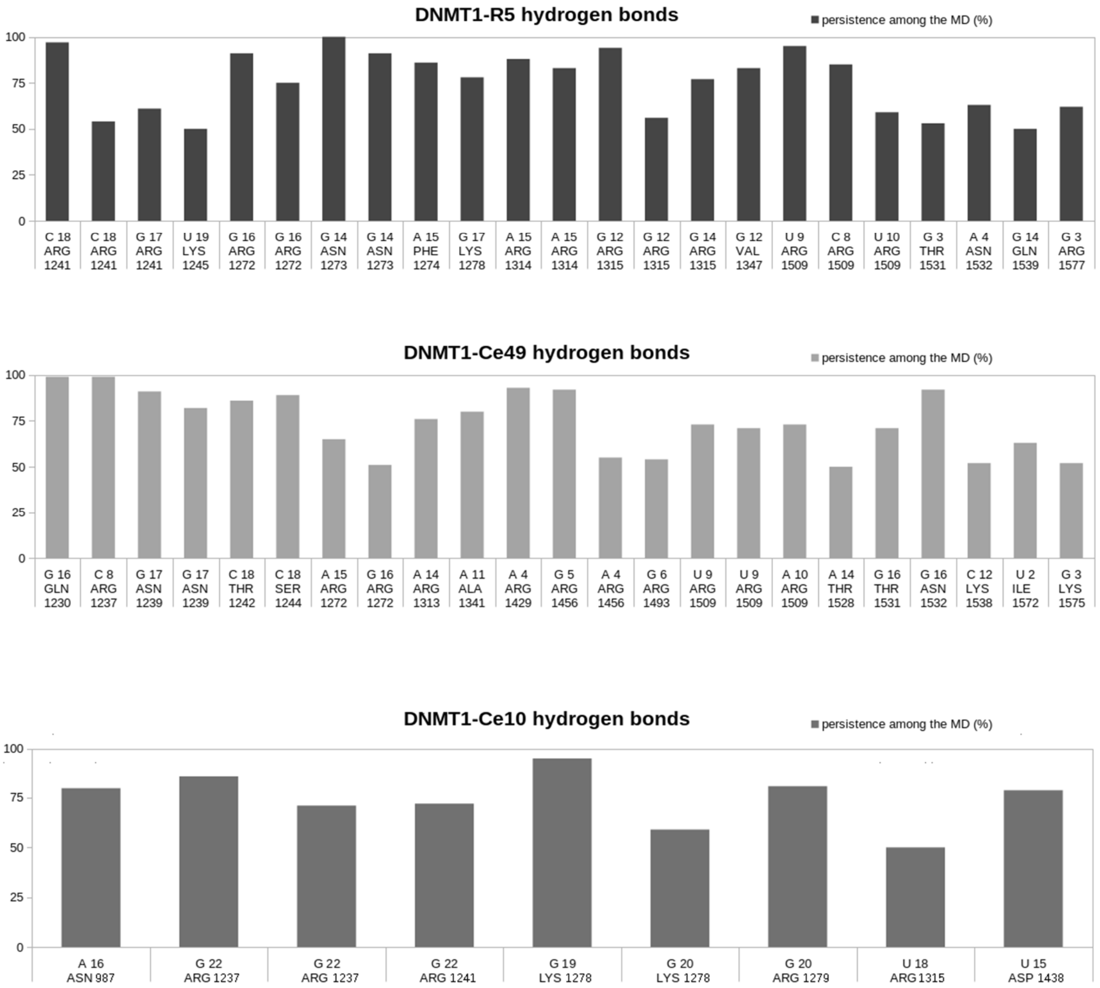

Supplementary Figure 13. Hydrogen bonds at R5- or aptamers-DNMT1 interfaces. Percentage of existence of hydrogen bonds at R5- or aptamers-DNMT1 interfaces, during the last 150 ns of simulation time performed with the amber Parmfsbc1 force field, of R5-DNMT1: top panel, Ce-49 sh-DNMT1: central panel, and Ce-10-2 sh-DNMT1: bottom panel. The couple of protein-nucleobase residues involved into each hydrogen bond are indicated at the x axis.

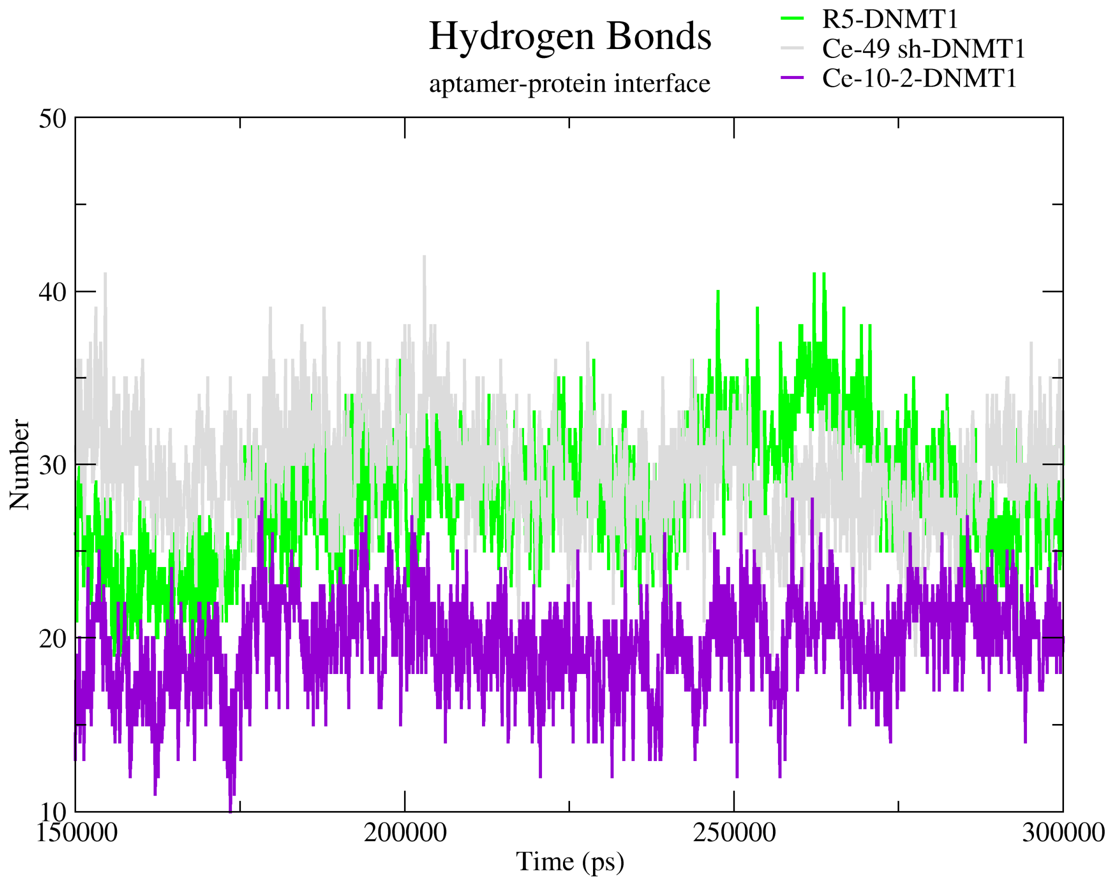

Supplementary Figure 14. Hydrogen bonds at complexes interfaces during the last 150 ns. Absolute number of R5- or aptamers-DNMT1 interfaces hydrogen bonds during the last 150 ns of simulation time performed with the amber Parmfsbc1 force field. The following color code was used: R5-DNMT1: green, Ce-49 sh-DNMT1: gray and Ce-10-2 sh-DNMT1: violet.

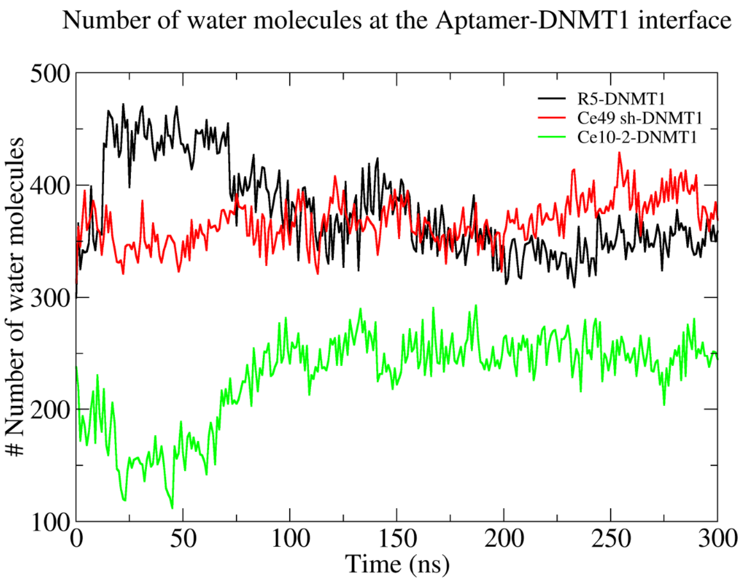

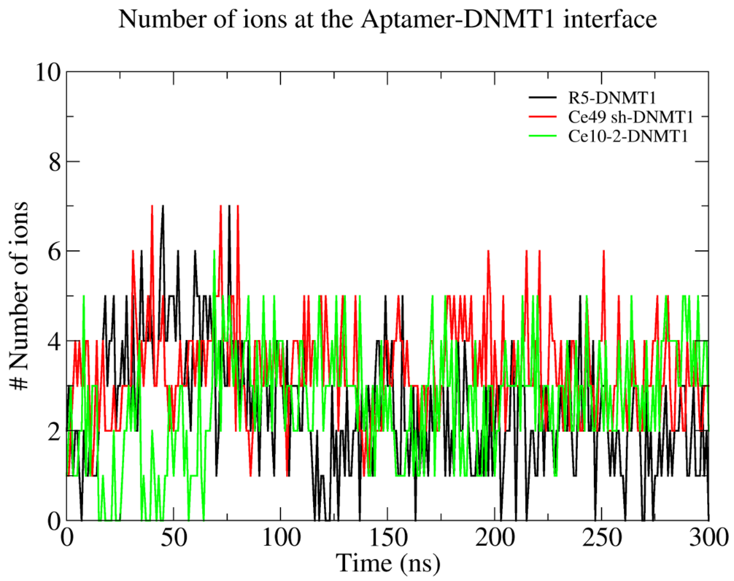

Supplementary Figure 15. **Water molecules and ions at the aptamer-DNMT1 interface.** Water molecules (top panel) and ions (bottom panel) at the aptamer-DNMT1 interface.along the molecular dynamic simulations performed with the amber parmfsbc1 force field. (water molecules mean values: 376, 368 and 230 for -R5, -Ce49 sh and -Ce10-2 sh with SD values: 40, 20 and 40 for -R5, -Ce49 sh and -Ce10-2 sh complexes respectively Ion mean values: 2, 3 and 3 for -R5, -Ce49 sh and -Ce10-2 sh with SD values: 1, 1 and 1 for -R5, -Ce49 sh and -Ce10-2 sh complexes respectively).

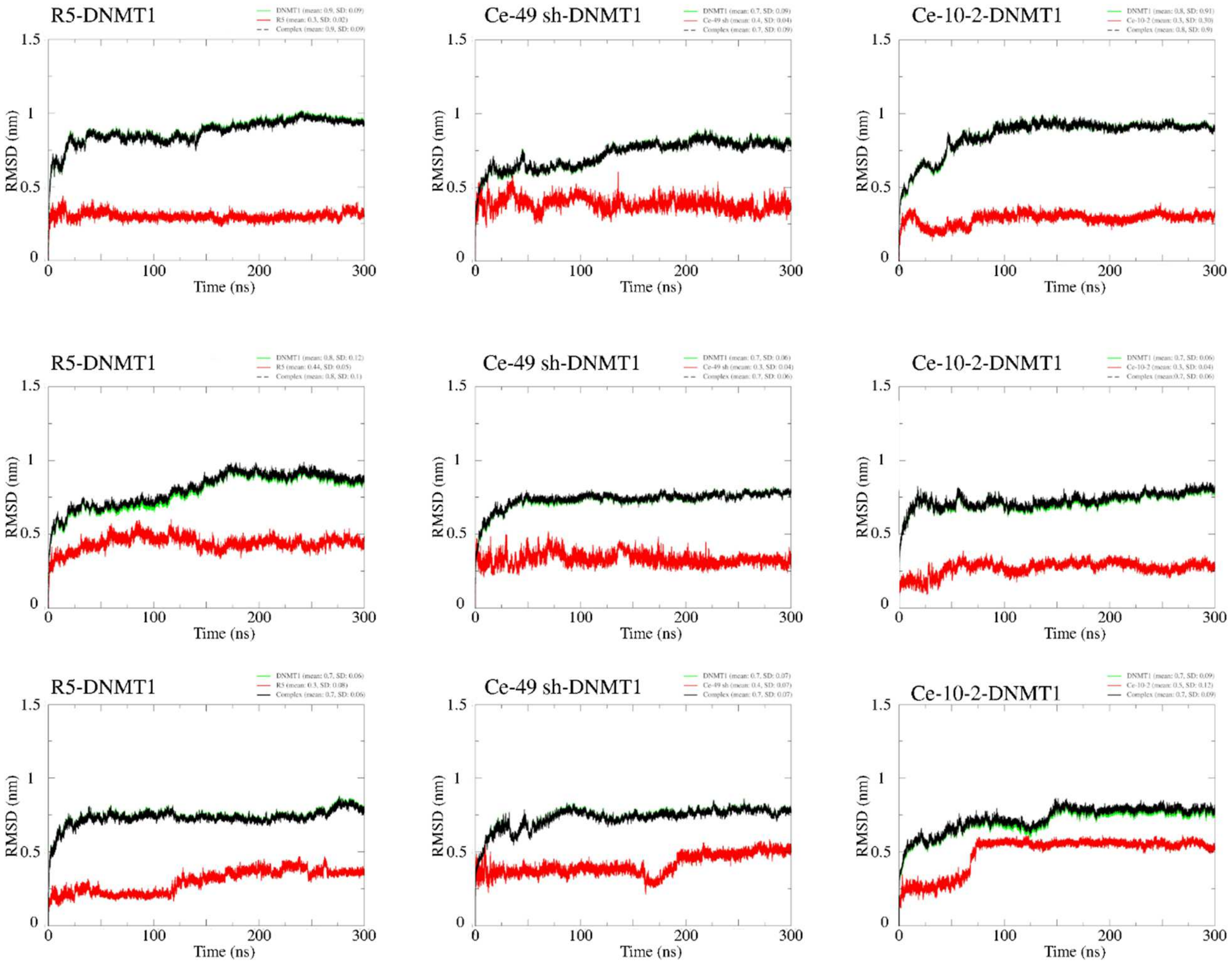

Supplementary Figure 16. RMSD profile. Time evolution of the RMSD values with respect to the starting models for the simulation replica performed with the OL15 force field parameters. The RMSD values have been computed considering the C alpha and C5' atoms of the protein and RNA, respectively. The following color code was used: overall complexes: black line, protein residues: green, RNA residues: red. Top panels: replica 1, middle panels: replica 2 and bottom panels: replica 3.

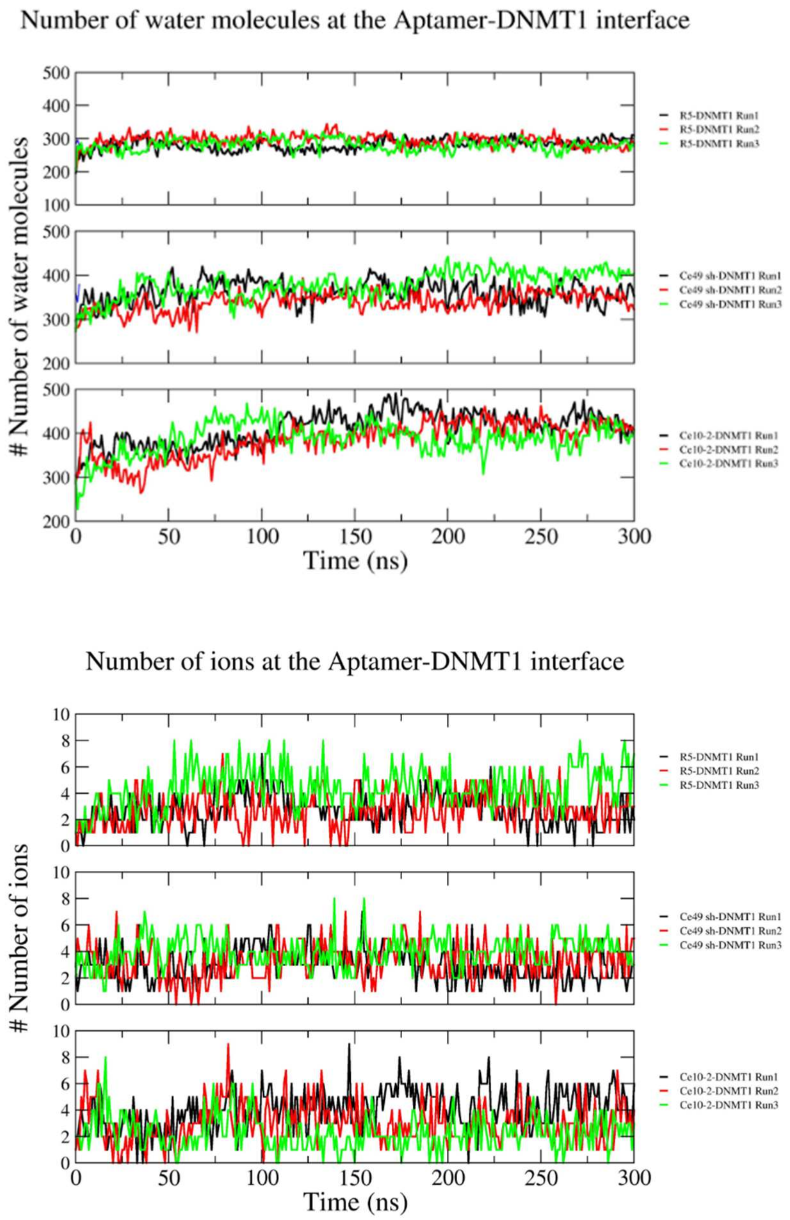

Supplementary Figure 17. Ions at the Aptamer-DNMT1 interface. Water molecules (top panel) and ions (bottom panels) at the Aptamer-DNMT1 interface along the three molecular dynamic simulations performed with the amber OL15 force field.

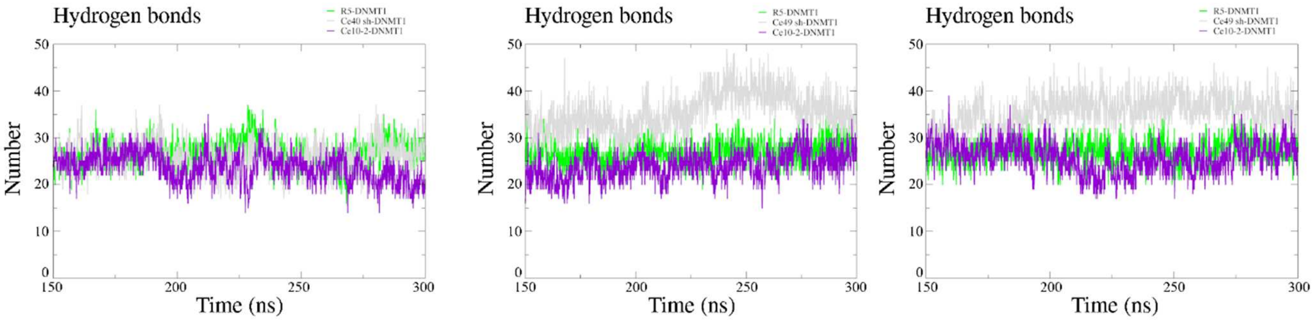

Supplementary Figure 18. Absolute number of R5- or aptamers-DNMT1 interfaces hydrogen bonds during the last 150 ns of simulation time performed with the amber OL15 force field. Left panel: replica 1, and. R5-DNMT1 (25.61, SD: 3.18), Ce49 sh-DNMT1 (mean: 25.7, SD: 3.33); Ce10-2 sh-DNMT1 (mean: 20.96, SD: 4.44). Middle panel: replica 2 Replica R2: R5-DNMT1 (25.13, SD: 6.09), Ce49 sh-DNMT1 (mean: 31.91, SD: 3.28); Ce10-2 sh-DNMT1 (mean: 19.75, SD: 6.36). Right panel replica 3: R5-DNMT1 (24.74, SD: 3.72), Ce49 sh-DNMT1 (mean: 33.53, SD: 5.23); Ce10-2 sh-DNMT1 (mean: 23.93, SD: 5.76). The following colour code was used: R5-DNMT1: green, Ce-49 sh-DNMT1: gray and Ce-10-2 sh-DNMT1: violet.

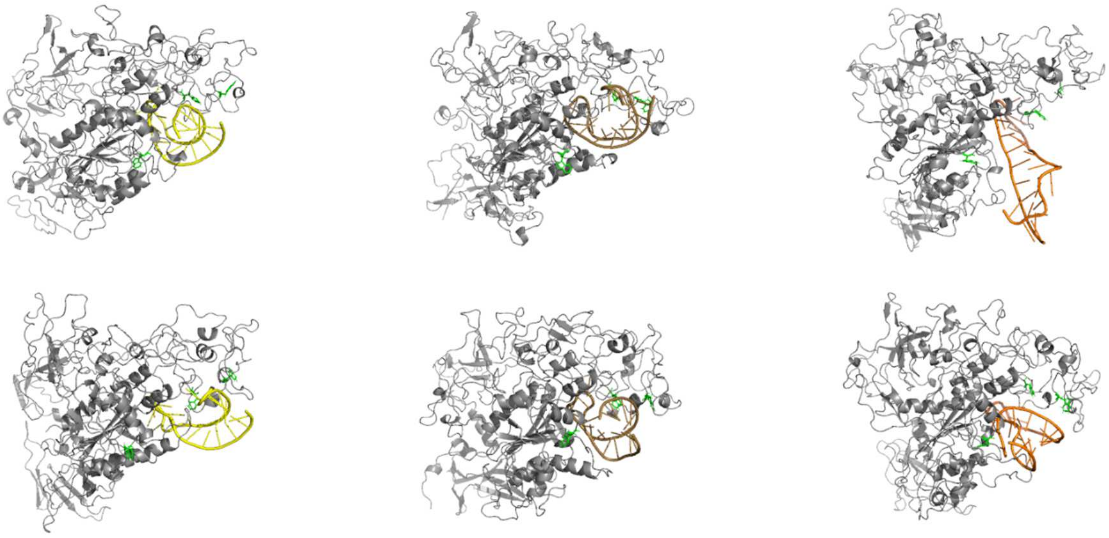

Supplementary Figure 19. Dynamic structures using a RMSD based clustering approach. Representative structures using a RMSD based clustering approach. Molecular dynamic representative structures of R5 of Ce-49 sh and Ce-10-2 sh aptamer-DNMT1 complexes derived using a RMSD based clustering approach. Top panel: molecular dymanic simulations performed with the parmbsc1 force field. Bottom panel: molecular dymanic simulations performed with the OL15 force field. DNMT1 protein is shown in cartoon and colored in gray. For aptamers, the following colour scheme was adopted: R5: yellow, Ce-49 sh: brown and Ce-10-2 sh: orange. The green sticks indicate tryptophan residues at the interfaces with the aptamers.

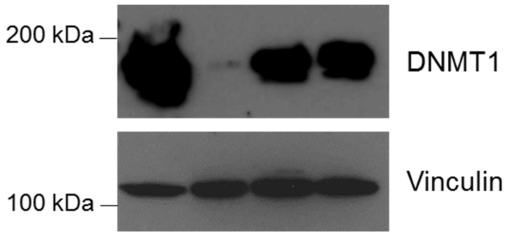

Supplementary Figure 20. DNMT1 levels. Levels of DNMT1 or Vinculin (used as a loading control) were analysed in cell extracts from indicated cell lines. Experiment was repeated independently two times with similar results.

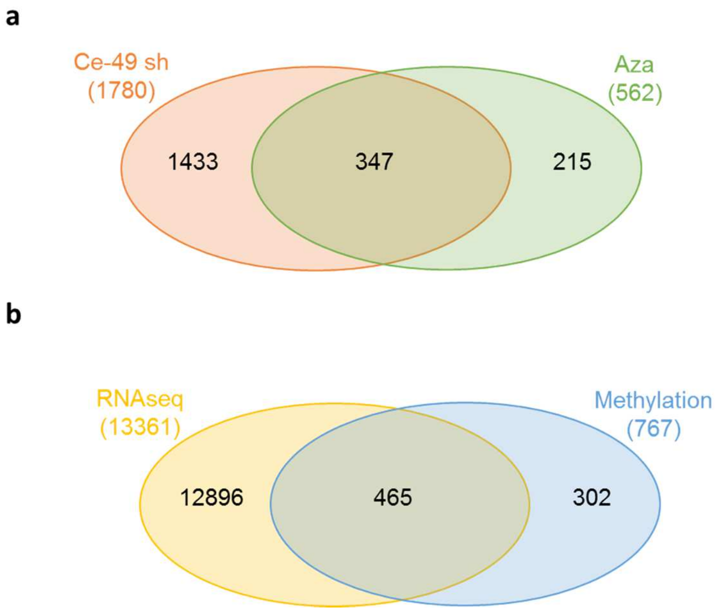

Supplementary Figure 21. Overlapping genes between treatments. (**a**) Overlap of genes differently expressed upon treatments with Ce-49 sh and 5-aza, (**b**) Overlap of genes with different methylated CG and the total genes detected in the RNA-seq experiment.

Supplementary Table 1. SELEX conditions

Round	RNA pool (pmol)	Protein (pmol)	RNA: protein ratio	Number of washes	Number of counter-selection
1	300	10	30:1	2	1
2	150	5	30:1	3	1
3	150	5	30:1	4	1

Supplementary Table 2. Hydrogen bond along the concatenated trajectory of molecular dynamic simulations performed with the OL15 force field.

Hydrogen bonds*	DNMT1 residue	Aptamer residue	Persistence*
**R5 DNMT1**	ARG1493	C7	46
	ARG1456	G5	39
	ARG1313	G14	36
	ARG1314	A15	34
	ARG1241	U19	30
	THR1528	G14	25
	ARG1509	C8	25
	ARG1493	G6	22
	THR1528	A15	21
	ARG1241	C18	20
**Ce 49 DNMT1**	SER1527	G13	72
	SER1244	C18	33
	ARG1509	C8	31
	ARG1509	U9	29
	ARG1241	U19	24
	ARG1493	G6	23
	LYS984	A11	22
	THR1528	A14	21
	ARG1313	C12	21
	ASP1438	C12	21
	ARG1272	A14	21
**Ce-10-2 DNMT1**	ARG1315	A17	26
	LYS984	U15	24
	ARG1493	G8	21
	LYS984	A16	21
	ARG1493	C9	21

* only those contacts occurred at least the 20% of the frames are reported.

Supplementary Methods

Calculation of K_D by MST

The K_D is calculated from the law of mass action, which is defined as

K_D=[A]*[L]/[AL]

where [A] is the concentration of free fluorescent molecule, [L] the concentration of free ligand and [AL] is the concentration of the complex of A and L. The free concentrations of A and L are [A]=[A_0]-[AL] and [L]=[L_0]-[AL], respectively. [A_0] is the known concentration of the fluorescent molecule and [L_0] is the known concentration of added ligand. This leads to a quadratic fitting function for [AL]:

$[AL]=1/2*(([A_0]+[L_0]+ K_D)-(([A_0]+[L_0]+ K_D)2-4*[A_0]*[L_0])1/2)$

The concentration of fluorescent molecule [A_0] is kept constant during the experiments and the concentration of ligand [L_0] is varied in a dilution series. The signal obtained in the measurement directly corresponds to the fraction of fluorescent molecules that formed the complex x=[AL]/[A_0], which can be easily fitted with the derived equation to obtain K_D.
