# Supplementary material for: Targeted systematic evolution of an RNA platform neutralizing DNMT1 function and controlling DNA methylation
Source: Nat Commun. 2023 Jan 6;14:99. doi: 10.1038/s41467-022-35222-4 (PMC9823104; doi:10.1038/s41467-022-35222-4)
Supplement: Supplementary file 3 — Description of Additional Supplementary Files [file 41467_2022_35222_MOESM3_ESM.pdf]

Description of Additional Supplementary Files

File Name: Supplementary Data 1
Description: Overlapping GO terms
