# Supplementary material for: Targeted systematic evolution of an RNA platform neutralizing DNMT1 function and controlling DNA methylation
Source: Nat Commun. 2023 Jan 6;14:99. doi: 10.1038/s41467-022-35222-4 (PMC9823104; doi:10.1038/s41467-022-35222-4)
Supplement: Supplementary file 5 — Reporting Summary [file 41467_2022_35222_MOESM5_ESM.pdf]

nature portfolio

Corresponding author(s): Di Ruscio A, Esposito CL, de Franciscis V, Kortylewski M

Last updated by author(s): Nov 17, 2022

Reporting Summary

Nature Portfolio wishes to improve the reproducibility of the work that we publish. This form provides structure for consistency and transparency in reporting. For further information on Nature Portfolio policies, see our Editorial Policies and the Editorial Policy Checklist.

Statistics

For all statistical analyses, confirm that the following items are present in the figure legend, table legend, main text, or Methods section.

n/a	Confirmed	
☐	☒	The exact sample size (*n*) for each experimental group/condition, given as a discrete number and unit of measurement
☐	☒	A statement on whether measurements were taken from distinct samples or whether the same sample was measured repeatedly
☐	☒	The statistical test(s) used AND whether they are one- or two-sided<br>*Only common tests should be described solely by name; describe more complex techniques in the Methods section.*
☒	☐	A description of all covariates tested
☐	☒	A description of any assumptions or corrections, such as tests of normality and adjustment for multiple comparisons
☐	☒	A full description of the statistical parameters including central tendency (e.g. means) or other basic estimates (e.g. regression coefficient) AND variation (e.g. standard deviation) or associated estimates of uncertainty (e.g. confidence intervals)
☐	☒	For null hypothesis testing, the test statistic (e.g. *F*, *t*, *r*) with confidence intervals, effect sizes, degrees of freedom and *P* value noted<br>*Give P values as exact values whenever suitable.*
☒	☐	For Bayesian analysis, information on the choice of priors and Markov chain Monte Carlo settings
☒	☐	For hierarchical and complex designs, identification of the appropriate level for tests and full reporting of outcomes
☒	☐	Estimates of effect sizes (e.g. Cohen's *d*, Pearson's *r*), indicating how they were calculated

Our web collection on statistics for biologists contains articles on many of the points above.

Software and code

Policy information about availability of computer code

Data collection	For RNA-Seq an Methylation Array, no code or software were used to collect data from any public or proprietary repository.

Data analysis

For BLI, data were exported from the BLItz Pro 1.2 software. For Microscale Thermophoresis, MO Control software v1.5.3 from Nanotemper Technologies was used for data acquisition and manipulation and Affinity analysis software v2.2.7 for data analyses and fitting.
The three-dimensional structure of the human DNMT1 protein was created by homology modelling with MODELLER 9.22 program using the mouse DNMT1 downloaded from the Protein Data Bank database (PDB: 4da4). MD simulations were run using the Parmbsc1 for DNA 25 with GROMACS 5.0.5.
The EPIC methylation array was analysed using the Bioconductor package RnBeads (v2.4.0) using the hg19 annotations. Background normalization method was set to "enmix.oob" with "swan" used for normalization. When setting up the sva covariates, only the "aptamers" designation was used. Differential methylation analysis was then performed comparing the respective "control" and "treatment" replicates. The heatmap output from RnBeads was used with minor adjustments for publication. For volcano plots, EnhancedVolcano (v1.4.0) was used. CpG sites with an adjusted FDR < 0.05 were set to dark red for plotting. CpG sites were annotated to their closest gene using the variable annotations provided with the array platform using a custom R script. For Gene Onology analysis, hypo-methylated CpG sites (with mean.diff >=0 value, FDR < 0.05) were selected, their HGCN gene ids extracted and analysed using Enrichr website (https://maayanlab.cloud/Enrichr/). The results reported herein correspond to the resulting "GO Biological Process" results.
The RNA-seq sequence data was aligned to the reference sequence (hg19) using STAR (ver2.7.9) with Gencode annotation database (v19). RNA-seq quantification was measured as raw count using featureCount feature from Subread package (ver2.0.3). The small RNA genes (length <200bps) and genes with low coverage (max expression less than 0.1 rpkm) are excluded for downstream different expression analysis. The different expression analysis of RNA-seq were applied using DESeq2 package (ver 1.26.0) with FDR<0.05 cutoff for different expressed gene. The heatmap is generated using R pheatmap (ver 1.0.12) on scaled expression data. The volcano plot is generated using R ggplot2 (ver 3.3.5).

For manuscripts utilizing custom algorithms or software that are central to the research but not yet described in published literature, software must be made available to editors and reviewers. We strongly encourage code deposition in a community repository (e.g. GitHub). See the Nature Portfolio guidelines for submitting code & software for further information.

Data

Policy information about availability of data

All manuscripts must include a data availability statement. This statement should provide the following information, where applicable:

- Accession codes, unique identifiers, or web links for publicly available datasets
- A description of any restrictions on data availability
- For clinical datasets or third party data, please ensure that the statement adheres to our policy

All our raw data, and a detailed data processing description have been submitted to GEO. Data will be made will be made publicly available upon manuscript acceptance under the accession ID number: GSE154471 (https://www.ncbi.nlm.nih.gov/geo/query/acc.cgi?acc=GSE154471; token: qdcnuomwxhaxfgt) for the in vitro data; GSE205655 (https://www.ncbi.nlm.nih.gov/geo/query/acc.cgi?acc=GSE205655; token: mbshgeayfxmrnql) for the in vivo data.

Human research participants

Policy information about studies involving human research participants and Sex and Gender in Research.

Reporting on sex and gender	n/a
Population characteristics	n/a
Recruitment	n/a
Ethics oversight	n/a

Note that full information on the approval of the study protocol must also be provided in the manuscript.

Field-specific reporting

Please select the one below that is the best fit for your research. If you are not sure, read the appropriate sections before making your selection.

☒ Life sciences ☐ Behavioural & social sciences ☐ Ecological, evolutionary & environmental sciences

For a reference copy of the document with all sections, see nature.com/documents/nr-reporting-summary-flat.pdf

Life sciences study design

All studies must disclose on these points even when the disclosure is negative.

Sample size

For the in vitro analyses , two or three biological replicates with two or three technical replicates were carried out without a sample size calculation based on previus studies. For in vivo study, sample size will be calculated considering an anticipated difference of at least 30% between the mean of two experimental groups (standard deviation of 12-15%), with a power of analysis between 90% and 95%, and an α-value of 0,01.

	and for the in vivo data five mice per each group were analysed.
Data exclusions	No data were excluded from the analyses
Replication	The experiments are representative of two or tree biological replicates as indicated in the figures legends.
Randomization	Mice were randomly allocated in the different groups.
Blinding	The investigators were not blinded. We considered that blinding was not relevant as the aim of the study is to assess the effect of the tested molecules in treated versus untreated mice or cell lines. On the other hand, our study does not include clinical trials for which blinding is more appropiate.

Reporting for specific materials, systems and methods

We require information from authors about some types of materials, experimental systems and methods used in many studies. Here, indicate whether each material, system or method listed is relevant to your study. If you are not sure if a list item applies to your research, read the appropriate section before selecting a response.

Materials & experimental systems

n/a	Involved in the study	
☐	☒	Antibodies
☐	☒	Eukaryotic cell lines
☒	☐	Palaeontology and archaeology
☐	☒	Animals and other organisms
☒	☐	Clinical data
☒	☐	Dual use research of concern

Methods

n/a	Involved in the study	
☒	☐	ChIP-seq
☒	☐	Flow cytometry
☒	☐	MRI-based neuroimaging

Antibodies

Antibodies used	Anti-DNMT1 antibody (Active Motif, #39204) Clone:60B1220.1
Validation	DNMT1 antibody was validated for WB by Active Motif (https://www.activemotif.com/catalog/details/39204/dnmt1-antibody-mab)

Eukaryotic cell lines

Policy information about cell lines and Sex and Gender in Research

Cell line source(s)	K562 cells, U937 cells, A549 and Calu-1 NSCLC cells were obtained from ATCC and grown in RPMI medium supplemented with 10% FBS (Sigma), in the absence of antibiotics, at 37 °C in a humidified atmosphere with 5% CO2.
Authentication	All cell line have been authenticated using Short Tandem Repeat (STR) analysis, that is one of the most useful methods in molecular biology to compare specific loci on DNA from two or more samples
Mycoplasma contamination	Negative for Mycoplasma contamination by polymerase chain reaction
Commonly misidentified lines (See ICLAC register)	Not Commonly misidentified lines were used

Animals and other research organisms

Policy information about studies involving animals; ARRIVE guidelines recommended for reporting animal research, and Sex and Gender in Research

Laboratory animals	NOD.Cg-Prkdcscid Il2rgtm1Wjl/SzJ (NSG mice) Strain #:005557. Each group consisted of 5 mice, matched by age and sex. Mice were maintained at standard temperature (20-23°C) with 30-70% humidity and a 12 light/12 dark cycle.
Wild animals	Study did not involve wild animals
Reporting on sex	Gender-based analysis was not relevant in our study as the aim of the study is to assess the effect of the tested molecules in treated versus untreated mice. Neverless mouse groups were matched by sex and no differences were detected between gender. Our findings does not apply to only one sex.

Field-collected samples	Study did not involve samples collected from the field
Ethics oversight	Study was performed within the approved IACUC protocol 10016 of City of Hope

Note that full information on the approval of the study protocol must also be provided in the manuscript.
